# Supplementary material for: Systematic Evaluation of Biotic and Abiotic Factors in Antifungal Microorganism Screening
Source: Microorganisms. 2024 Jul 10;12(7):1396. doi: 10.3390/microorganisms12071396 (PMC11279232; doi:10.3390/microorganisms12071396)
Supplement: Supplementary file 1 [file microorganisms-12-01396-s001.zip › microorganisms-3083216-supplementary.pdf]

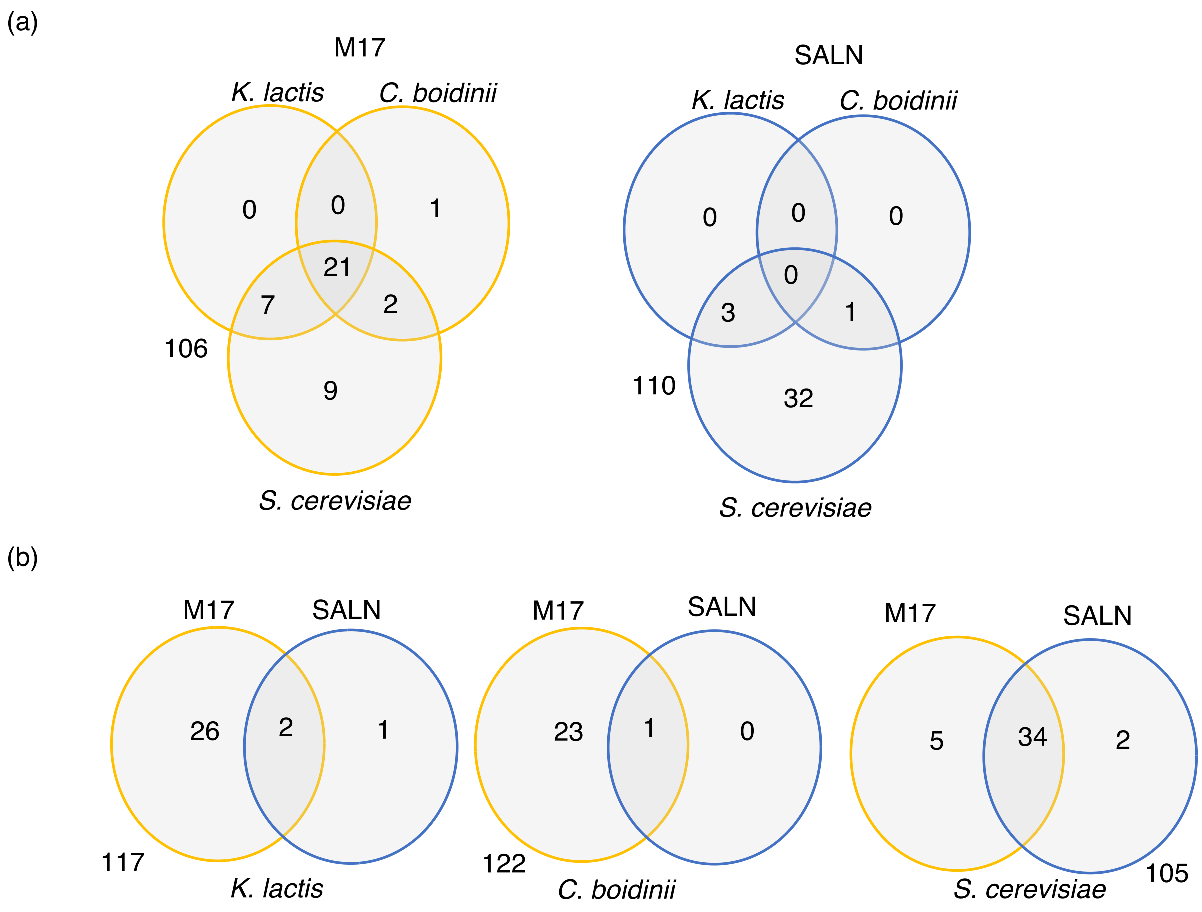

Supplementary Figure S1: Venn diagram showing the number of isolates that were isolated on LM17 exhibiting antifungal effect (a) against the three target yeasts in each culture medium and (b) in two culture media for each target yeast.

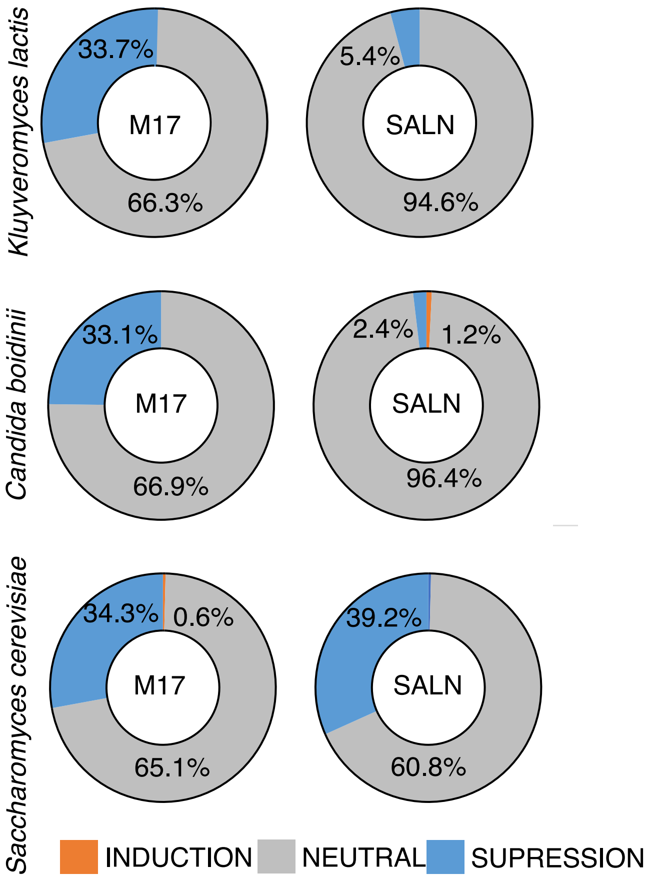

Supplementary Figure S2: Pie charts showing the percentage of interactions that induce or suppress antifungal activity in the second set of experiments. Most of the interactions were neutral. The frequency of interaction-mediated induction of the antifungal effect is significantly less than interaction-mediated suppression for all three yeast targets (Log likelihood test, Chi-square Prob < 0.0001). N (M17, SALN) = 166

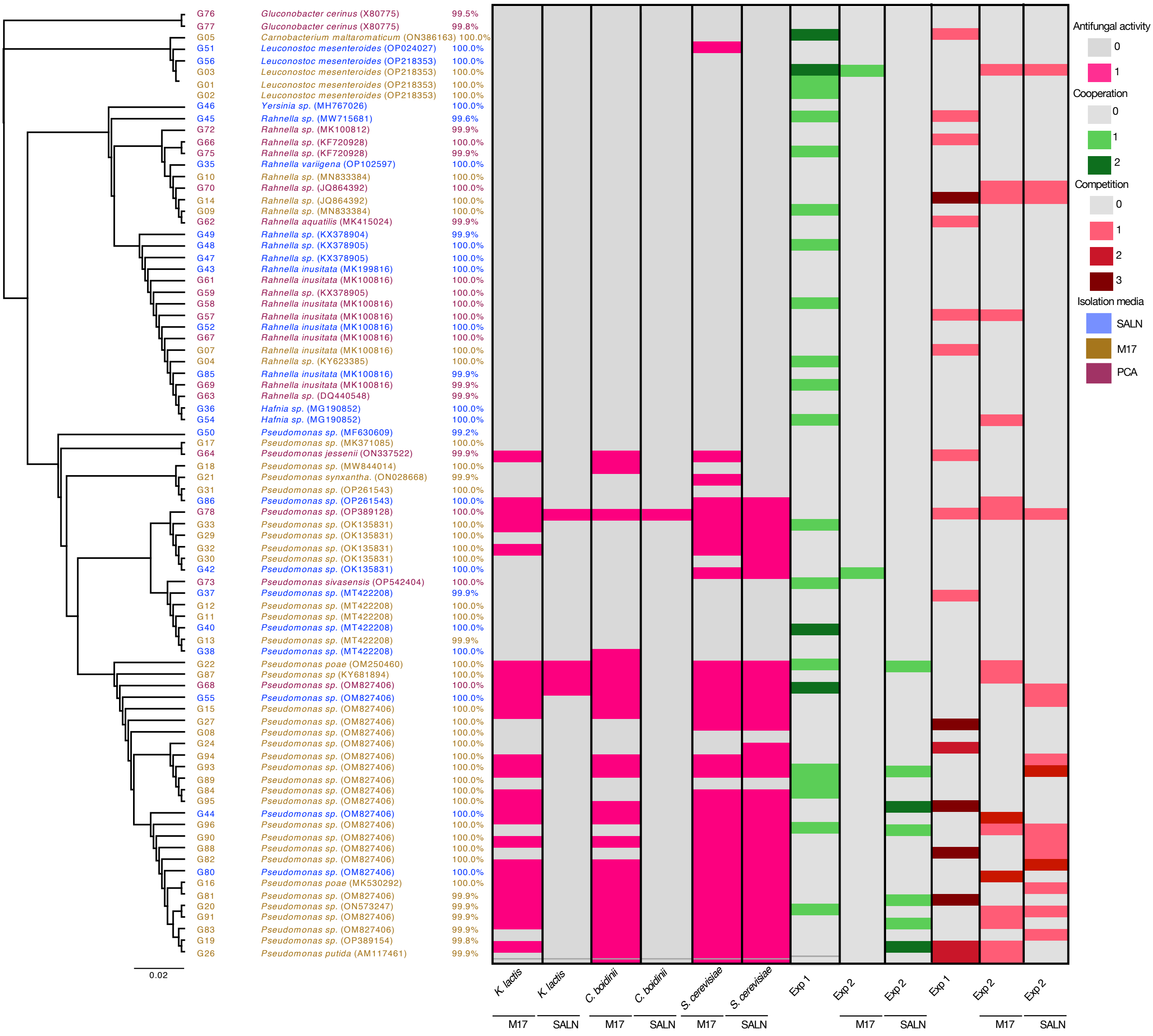

Supplementary Figure S3: Phylogenetic tree and heat map of antifungal, cooperation, and competition scores maple sap isolates. The tree is based on partial 16S rRNA gene sequence of each isolate. The neighbor-joining method, Kimura 2-parameter (1000 bootstrap replicates) was used to construct the tree. Distance units represent the number of base substitutions per site. The isolate name, the BLAST results for the closest cultured strain, and the percentage of pairwise identity are identified on the left. Isolates are color coded on the basis of their isolation media. Additionally, the heatmap on the right provides information on their antifungal activity, cooperative behavior, and competitive behavior.

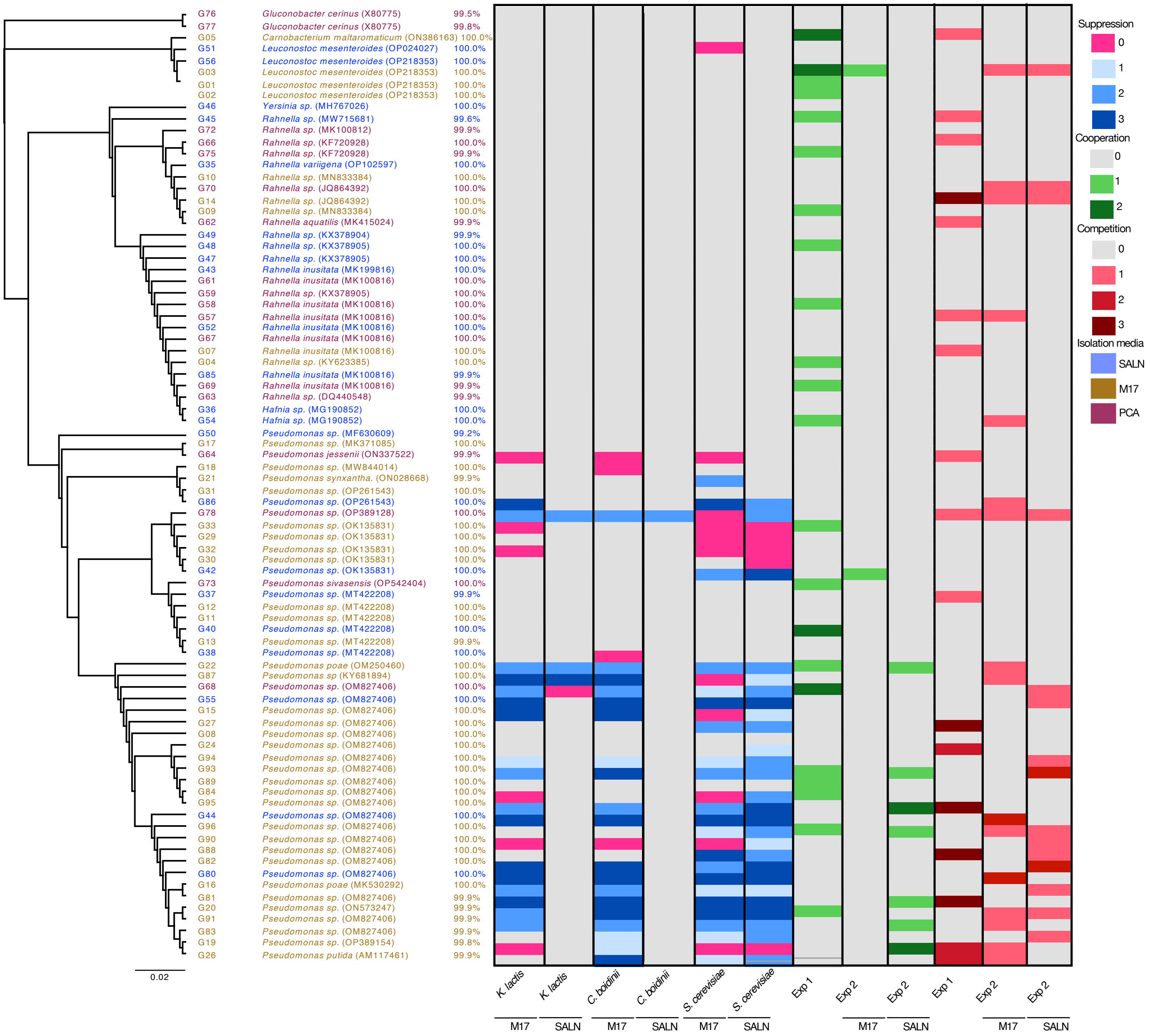

Supplementary Figure S4: **Phylogenetic tree and heat map of antifungal suppression, cooperation, and competition scores of 82 maple sap isolates**. The tree is based on partial 16S rRNA gene sequence of each isolate. The neighbor-joining method, Kimura 2-parameter (1000 bootstrap replicates) was used to construct the tree. Distance units represent the number of base substitutions per site. The isolate name, the BLAST results for the closest cultured strains, and the percentage of pairwise identity are identified on the left. Isolates are color coded on the basis of their isolation media. The heat map on the right provides information on the suppression score, cooperative behavior, and competitive behavior.
